# Development of a Scalable High-Power LED-Based Inspection Platform for Correlative Photoluminescence and Electroluminescence Mapping of LED Chips

**DOI:** 10.3390/mi17080949

**Published:** 2026-08-10

**Authors:** Shang-Ping Ying, Bing-Mau Chen, Phan Van Kiet, Chen-Feng Lin

**Affiliations:** Department of Semiconductor and Electro-Optical Technology, Minghsin University of Science & Technology, 1, Xinxing Road, Xinfeng, Hsin-Chu 30401, Taiwan; phankiet24102001@gmail.com (P.V.K.); as0970704158@gmail.com (C.-F.L.)

**Keywords:** photoluminescence (PL) mapping, electroluminescence (EL) mapping, LED chip inspection, normal-collection geometry, oblique-collection geometry

## Abstract

In this work, we developed a measurement platform that employs high-power LEDs as excitation sources in conjunction with a charge-coupled device to perform spatially resolved photoluminescence (PL) and electroluminescence (EL) mapping of commercial LED chips. Two optical configurations (i.e., normal and oblique incidence) were designed to directly compare PL and EL distributions. Unlike conventional laser-based systems, high-power LEDs provide a cost-effective, compact, and scalable solution for PL mapping and provide a broad area of illumination, making them highly suitable for rapid chip-level inspection with strong potential for future wafer-scale scaling. The PL spectra obtained under high-power LED excitation exhibited strong correspondence with the EL spectra. Spatial mappings revealed key emission features, such as metallic contacts, defect-related spots, and surface contamination. The oblique-collection geometry yielded a more intense PL signal and clearer localized emission variations relative to the normal-collection geometry. These findings demonstrate that high-power LED-based PL mapping represents a practical and noninvasive alternative to EL, providing a reliable and scalable pathway for optical inspection of LED chips in both research and industrial applications.

## 1. Introduction

GaN-based light-emitting diodes (LEDs) have been widely applied in solid-state lighting, displays, and optoelectronic devices because of their high efficiency and long operational lifetime. The structural quality and defect distribution within LED chips must be determined to optimize device performance, given that point defects, V-pits, and indium clustering considerably affect carrier recombination processes and emission [1]. Conventional characterization techniques, such as X-ray diffraction, transmission electron microscopy, and scanning electron microscopy, have been widely applied to investigate crystalline defects and structural features. However, these methods are often destructive, time-consuming, and expensive, making them unsuitable for rapid or large-scale wafer inspection. Photoluminescence (PL) has emerged as a fast, contactless, and nondestructive alternative that can be performed directly on epitaxial wafers before electrode fabrication, enabling investigators to detect recombination pathways and evaluate carrier lifetimes with high sensitivity [2,3,4,5]. Notably, PL is not limited by the small electrode dimensions or fragile nature of wafers, which can hinder electrical probing of microLEDs [6,7]. Thus, PL provides a scalable solution for wafer-level inspection in industrial production. Moreover, correlations between PL and electroluminescence (EL) have demonstrated that PL can predict electrical characteristics, bridging optical inspection with device-level performance evaluation [8,9].

PL mapping, a powerful extension of conventional PL techniques, leverages these advantages to facilitate investigation of LED chips and wafers. PL mapping enables spatially resolved visualization of luminescence characteristics across large areas, revealing localized nonradiative defects, carrier leakage paths, and indium composition fluctuations in InGaN-based structures [1,10]. Advanced micro-scale techniques, such as micro-photoluminescence (PL) mapping [10] and near-field scanning optical microscopy NSOM mapping [11,12], have been instrumental in investigating carrier localization and defect-related efficiency mechanisms in InGaN LED and microLEDs [10,11,12,13]. This method provides highly valuable wafer-level information for process monitoring, allowing investigators to identify defective regions before device fabrication. Unlike EL, which requires electrical contacts, PL mapping relies only on optical excitation and detection with a charge-coupled device (CCD), making this method noninvasive, rapid, and scalable to full-wafer inspection. Furthermore, PL imaging has been widely employed to correlate optical signatures with electrical performance, such as I–V characteristics and efficiency droop behavior, reinforcing its role as a predictive diagnostic tool for both microLEDs and conventional LEDs in industrial applications [7,8,9].

PL measurements have traditionally relied on laser excitation due to their narrow spectral linewidth and high intensity. Laser-based PL systems provide high spatial resolution and localized excitation, making them particularly suitable for spectroscopic characterization and defect analysis. However, laser-scanning and LED-based PL systems are designed for different measurement objectives and therefore involve different trade-offs in spatial resolution, inspection throughput, excitation area, and system complexity. By contrast, high-power LEDs represent a compact and cost-effective excitation source with a broader illumination area, making them particularly suitable for wide-field PL mapping when combined with CCD-based detection. Pantova et al. developed an ultraviolet LED-based PL measurement system for GaN samples, employing optical filters and a CCD scheme to successfully capture defect-related luminescence signatures [14]. Similarly, LED array excitation systems have enabled lifetime imaging of semiconductors, providing both temporal and spatial information on carrier dynamics with relatively simple instrumentation. These developments indicate that high-power LEDs can serve as an effective alternative excitation source for wide-field PL mapping, expanding the practical application of PL characterization in wafer- and chip-scale investigations while offering a pathway toward nondestructive, large-area, and cost-efficient optical inspection in both research and industrial settings.

Conventional laser-based scanning PL systems provide sub-micron spatial resolution and high local excitation density; however, they inherently suffer from limited throughput due to point-by-point raster scanning, often requiring several minutes per chip. In contrast, the proposed wide-field high-power LED platform prioritizes high-throughput functional inspection by illuminating the entire chip footprint simultaneously. Combined with background-suppression optics (long-pass filtering and optimized collection geometry), this setup achieves sufficient optical contrast and dynamic range within an exposure time of only a few hundred milliseconds (acquisition time < 1 s), making it highly scalable for rapid chip-level evaluation without requiring complex laser-scanning optics. However, most previously reported LED-excited PL systems primarily focused on luminescence detection or defect visualization, while quantitative spatial correlation between photoluminescence and electroluminescence distributions has received comparatively limited attention. Furthermore, systematic comparisons between different PL collection geometries have rarely been reported. Unlike previous UV-LED-based PL systems, the present work establishes a correlative PL–EL mapping platform incorporating both normal-collection and oblique-collection geometries. The proposed architecture enables direct pixel-by-pixel comparison between PL and EL distributions and provides quantitative evaluation through R^2^ analysis. To the best of our knowledge, such a dual-geometry LED-excited PL platform for systematic PL–EL correlation analysis across blue, green, and red LED chips has not been previously reported. In this work, we propose a high-power LED-excited PL–EL mapping platform for investigating the spatial correlation between optically and electrically generated luminescence in commercial LED chips. Two complementary collection geometries, namely normal-collection and oblique-collection configurations, were developed to systematically evaluate the influence of optical collection architecture on PL–EL correspondence. By combining wavelength-selective excitation, long-pass filtering, spatially resolved PL mapping, and quantitative pixel-by-pixel R^2^ analysis, the proposed platform provides a practical framework for correlating spatial optical emission features with electrical emission characteristics from purely optical measurements, without requiring electrical contact prior to packaging. In addition, the applicability of the proposed methodology is demonstrated using commercial blue, green, and red LED chips, enabling a systematic comparison of PL–EL correlation across different emission wavelengths. This approach offers a low-complexity, non-destructive pathway for chip-level LED performance evaluation, providing a scalable technical foundation that can be extended toward future wafer-scale optical screening.

**Figure 1:**
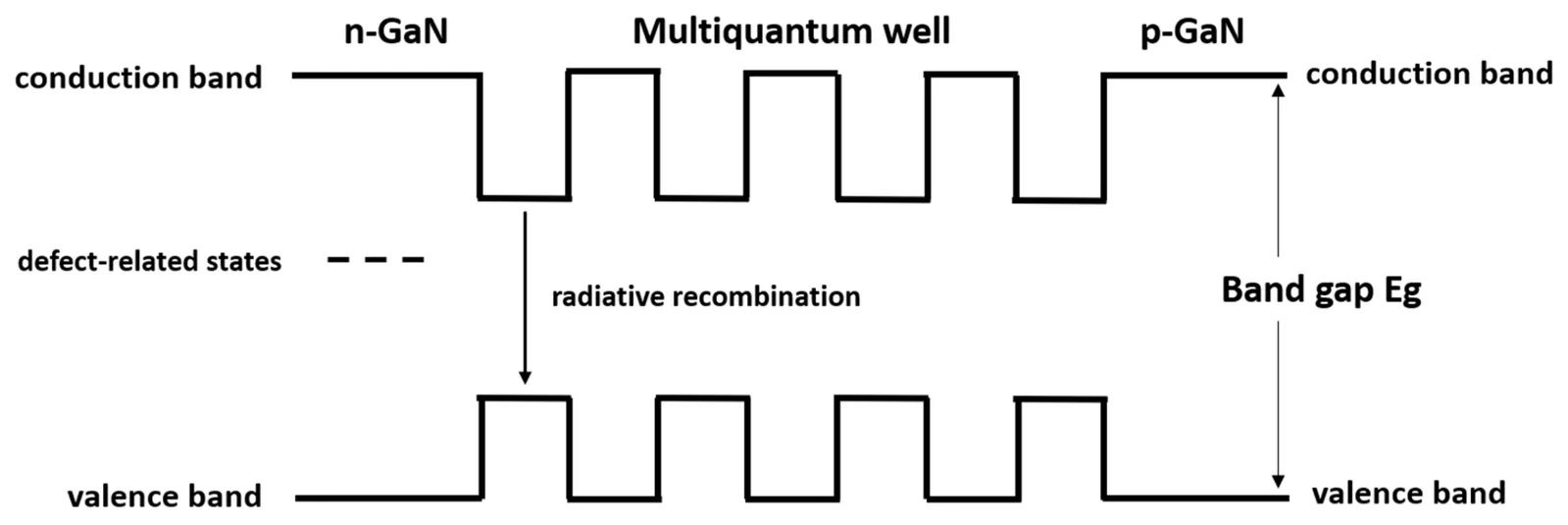
Schematic energy band diagram of a GaN-based multiple quantum well (MQW) light-emitting diode.

To establish the physical context for the empirical approach adopted in this work, a generic schematic of the energy band structure of a typical GaN-based multiple quantum well (MQW) light-emitting diode is presented in Figure 1. This schematic is not intended to represent the exact epitaxial structure or proprietary design of the commercial LED chips studied here; instead, it serves to illustrate the fundamental radiative recombination mechanisms common to GaN-based LEDs. As conceptually depicted, both electroluminescence, driven by electrical carrier injection, and photoluminescence, driven by optical excitation, ultimately result in electron–hole recombination within the multiple quantum wells, which constitutes the primary emission pathway. This shared recombination process provides the physical basis for the strong spectral and spatial correlation between PL and EL mappings investigated in this study. In addition, the schematic offers a qualitative framework for understanding secondary emission pathways, such as defect-related recombination, which are relevant to the weak and broad yellow luminescence observed in certain devices (e.g., the blue LED chip). By introducing this conceptual band diagram, we aim to provide readers with a clear physical reference that supports the interpretation of the PL–EL correlation results presented in the following sections.

## 2. Materials and Methods

**Figure 2:**
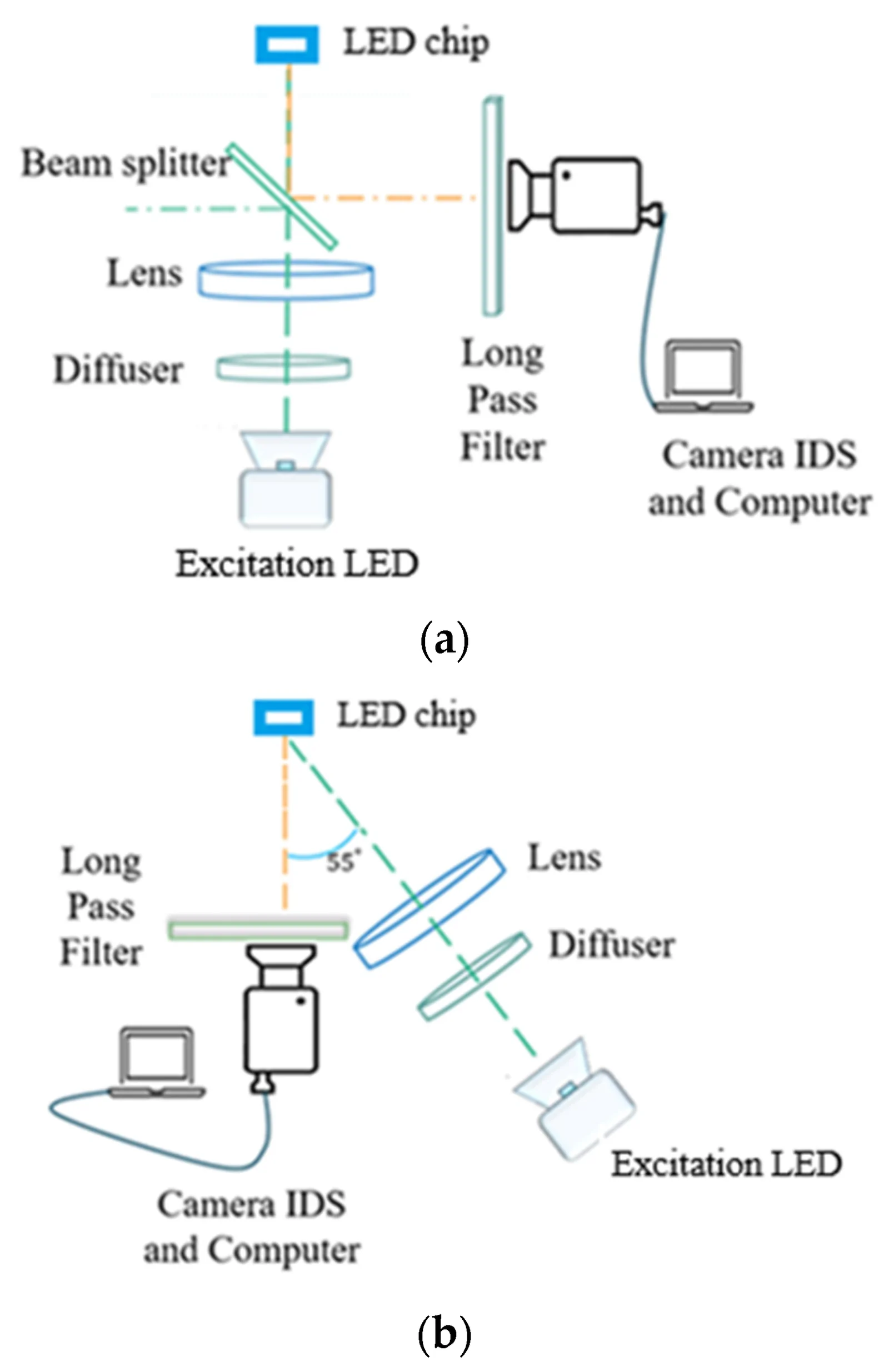
Optical measurement configurations. (**a**) Normal-collection geometry. (**b**) Oblique-collection geometry.

In this study, two optical measurement configurations were developed to perform spatially resolved PL and EL mapping of LED chip samples through high-power LED excitation and CCD detection. The first configuration is the normal-collection geometry, illustrated in Figure 2a. For this configuration in this study, the excitation LED was positioned in front of a diffuser and a plano-convex lens with a focal length of 65 mm. A 50:50 nonpolarizing beam splitter was placed 30 mm in front of the LED chip sample, with the total lens-to-sample separation maintained at 60 mm. The beam splitter was oriented at 45° to the incoming excitation beam, transmitting half of the light forward to illuminate the sample while reflecting the remaining half laterally. In this setup, luminescence emitted from the sample passed back through the beam splitter and was redirected toward the CCD system, which detected the luminescence after it traversed a long-pass filter placed directly in front of the lens to block reflected excitation light and environmental stray light. Quantitatively, this coaxial setup imposes a theoretical two-way optical transmission efficiency of T = 0.5 × 0.5 = 25% (corresponding to a 75% power loss or a ~6 dB optical attenuation along the measurement path) prior to detection. EL mapping was obtained under the same conditions through the application of a driving current to the LED chip sample, allowing the CCD to directly capture the EL distribution under normal-incidence conditions.

The second optical measurement configuration is the oblique-collection geometry, illustrated in Figure 2b. For this configuration in this study, the excitation LED was positioned at an angle of 55° relative to the surface normal of the LED chip sample. This geometry was deliberately selected to minimize the effects of direct reflections on the CCD optics and ensure the uniformity of excitation across the sample surface. In this setup, the output of the excitation LED was first homogenized by a diffuser and subsequently focused by a plano-convex lens with a focal length of 60 mm, forming a convergent illumination profile incident at the oblique angle. We aligned an identical CCD system to that used in the first configuration along the normal line of the LED chip sample, with a long-pass filter placed directly in front of the lens to suppress reflected excitation light and environmental stray light, ensuring that only the luminescence signal was captured. This setup also allowed for EL mapping by driving the LED chip with an injection current and directly capturing the EL with the CCD. This oblique-incidence geometry effectively reduces the likelihood of excitation light reflecting into the CCD, increasing the signal-to-noise ratio in PL mapping measurements.

In both optical measurement configurations, an IDS U3.3800CP-M-GL Rev.2 camera with an OPT-10M10-65A-1C machine vision lens was used to achieve high-resolution detection. The lens is characterized by a fixed working distance (WD) of 65 mm and a 1.0× magnification, delivering a maximum measurable field of view (FOV) diameter of approximately 17.6 mm. This system can deliver a total resolution of 20.44 megapixels (5120 × 2160 pixels), with a 2.4 µm pixel pitch on a 13.286 mm × 8.8861 mm CMOS sensor. The C-mount interface ensures broad compatibility with standard optical lenses, offering modular flexibility for different measurement requirements. The system’s high-resolution capability enables precise defect inspection and detailed luminescence mapping of variously sized LED chips. Our characterization platform utilizes two distinct modes of operation to fully assess the LED chips: spectral measurements and spatial intensity mapping. The spectral measurements were performed using an optical fiber coupled to a miniature spectrometer (SM445, Spectral Products, Putnam, CT, USA). The collection optics were configured to achieve an integrated, whole-chip measurement: specifically, while the optical fiber’s core diameter is approximately 600 µm and the chip size is typically 1000 µm in size, the light emitted from the entire chip area was collected and coupled into the fiber. This process yields a single spectrum that represents the average emission characteristics of the whole device but inherently lacks spatial resolution. This whole-chip fiber spectroscopy mode is specifically designed for rapid verification of the average peak wavelength and spectral profile against chip specifications. While it does not resolve localized spectroscopic features, spatial non-uniformities and defect distributions are complementarily resolved through the high-resolution CCD intensity mapping mode. This dual-mode strategy prioritizes throughput and operational simplicity, aligning with the primary goal of rapid pre-packaging inline screening. Conversely, the spatial intensity mapping was conducted using the CCD camera setup. This mode captures the light intensity across the chip area without spectral resolution (by utilizing long-pass to isolate the emission band), producing the two-dimensional intensity matrix that provides the spatial information about non-uniformities and defects crucial for our linear correlation coefficient (R^2^) correlation analysis. To establish a consistent and reproducible evaluation baseline across all chip types (blue, green, and red) and collection configurations (normal and oblique), the camera integration time was kept strictly constant at 7500 ms for all PL spatial mapping measurements and 150 ms for all EL spatial mapping measurements, ensuring a high dynamic range without sensor saturation. The combination of normal- and oblique-incidence geometries provides a versatile platform that balances excitation uniformity, reflection suppression, and direct comparability with EL measurements, offering a low-complexity, scalable, and non-destructive pathway for chip-level performance evaluation and future wafer-scale optical screening applications.

**Figure 3:**
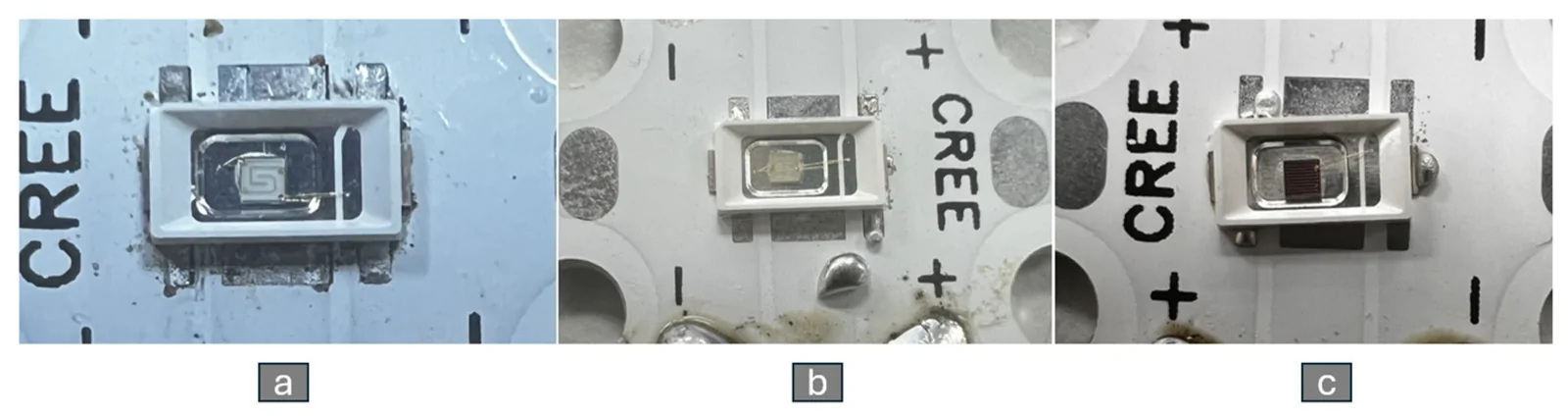
Surface mount device package samples with (**a**) blue chip, (**b**) green chip and (**c**) red chip.

We obtained three commercial LED chip samples in blue, green, and red from different manufacturers for use as test samples. Each chip had dimensions of approximately 1000 µm × 1000 µm and was mounted onto a surface mount device (SMD) package with die-attach adhesive, as illustrated in Figure 3. Table 1 summarizes the detailed specifications of the commercial LED chip samples, including the peak wavelength, and chip dimensions. Electrodes on the chip surface were connected to the leads of the SMD package with gold wires, facilitating current injection. No encapsulation was applied to the SMD package during the experiments to prevent the packaging materials from affecting the measurements, increasing the accuracy of our evaluation of the EL and PL properties of the LED chips.

We employed high-power LEDs as excitation sources to investigate the PL and EL mappings of commercial LED chips. In PL measurements, the excitation wavelength must be carefully chosen according to the band gap of the target material to ensure efficient carrier generation. Using photons with energy far above the band gap excites carriers to higher conduction states, which rapidly relax through nonradiative recombination or defect-related processes, reducing spectral specificity [2,3]. On the other hand, excitation wavelengths too close to the band edge may result in insufficient absorption, surface-dominated recombination, or thermal effects, thereby limiting PL signal intensity [4,5]. In our experiments, we selected high-power LEDs with peak wavelengths of 375, 408, and 530 nm as excitation sources for the blue (450 nm), green (535 nm), and red (659 nm) chips, respectively, ensuring sufficient excess photon energy to efficiently generate carriers while preserving the reliability and spectral fidelity of PL characterization. Each excitation LED was operated at an electrical input power of 2 W, and a beam diffuser/homogenizer was incorporated into the illumination path to ensure uniform excitation across the 17.6 mm field of view (FOV) provided by the OPT-10M10-65A-1C lens.

In both optical measurement configurations, a long-pass filter was positioned between the LED chip sample and the CCD. To block background and reflected excitation light, this filter was placed as close as possible to the CCD, ensuring that only the luminescence emitted from the LED chip sample was recorded (Figure 2). Long-pass filters are optical elements that block shorter wavelengths while transmitting longer wavelengths, with the cut-on wavelength selected according to the excitation source and emission band of the test sample. Given the selected excitation sources, a 420 nm long-pass filter was employed for the blue chip (450 nm emission) excited by the 375 nm high-power LED, a 500 nm long-pass filter for the green chip (535 nm emission) excited by the 408 nm LED, and a 600 nm long-pass filter for the red chip (659 nm emission) excited by the 530 nm LED. This configuration effectively suppressed the reflected excitation light, ensuring that the PL signal recorded from each sample corresponded solely to the emission band of interest. This approach ensured the accuracy of both our spectral and imaging measurements.

## 3. Results

**Figure 4:**
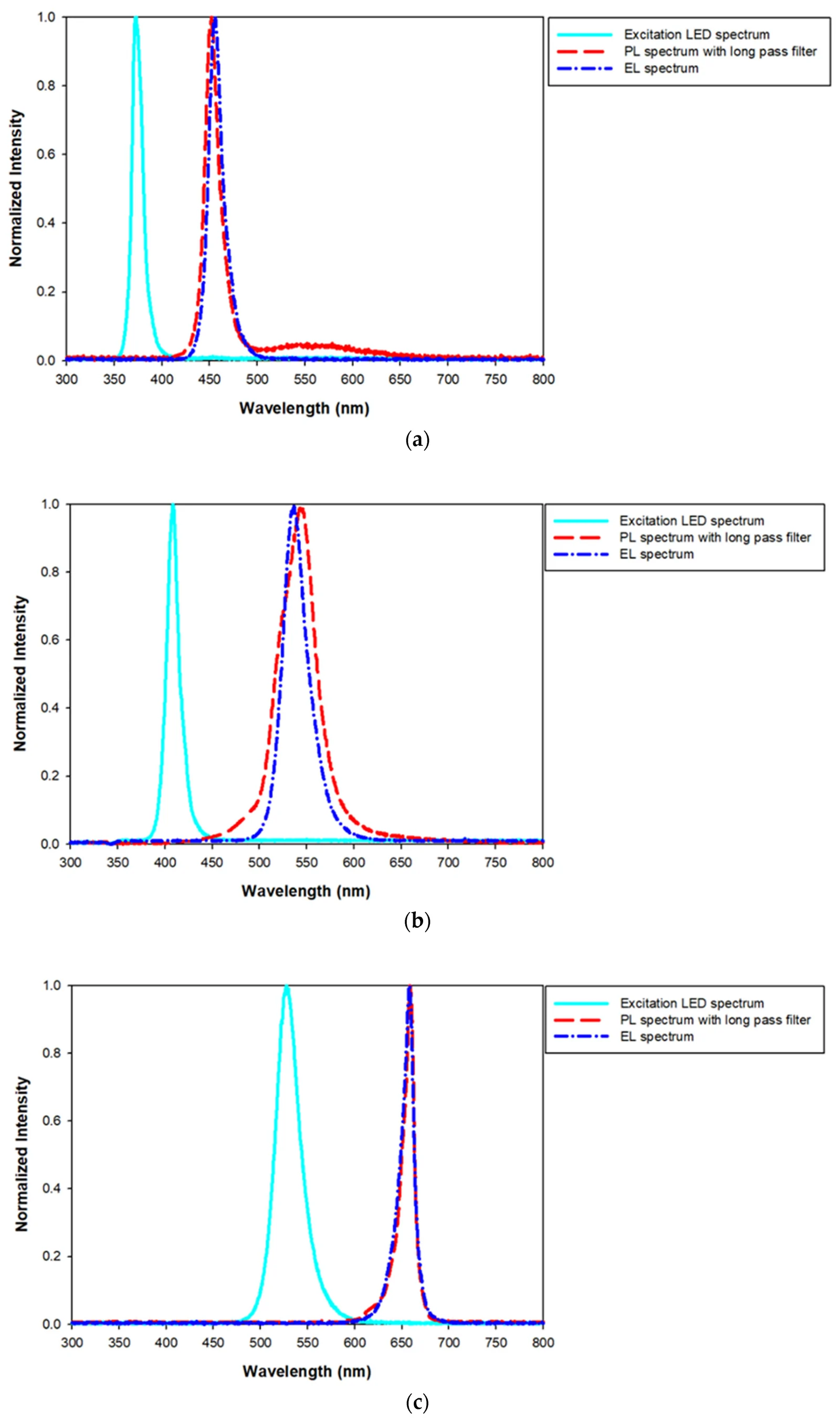
Photoluminescence (PL) and electroluminescence (EL) spectra for the (**a**) blue, (**b**) green, and (**c**) red LED chips, along with the respective excitation LED spectra.

Figure 4a–c display the PL spectra under high-power LED excitation and the EL spectra at an injection current of 0.15 A for the blue, green, and red LED chips. The observed PL spectra include luminescence components originating from the EL process, as evidenced by the spectral overlap and similarities in peak positions and emission profiles between the PL and EL findings. This observation suggests that the PL response not only reflects optical excitation but also probes intrinsic radiative recombination pathways that are activated under electrical injection, thereby revealing a strong correlation between PL and EL characteristics. In Figure 4a, two emission features are observed in the PL spectrum of the blue LED chip: a dominant peak centered at approximately 450 nm that coincides with the EL emission, and a weaker, broad band around 570 nm. This additional emission is attributed to defect-related radiative recombination in GaN-related layers outside the MQW active region and is commonly referred to as yellow luminescence in GaN-based materials [15]. The PL spectrum of the green LED chip in Figure 4b exhibits a distinct emission peak near 540 nm that generally aligns with the EL spectrum and represents the characteristic radiative transition of the device. Minor differences in peak position and spectral width between PL and EL are attributed to carrier-density-dependent effects in the InGaN/GaN MQW structure, including reduced quantum-confined Stark effect and band-filling under electrical injection. As shown in Figure 4c, the PL spectrum of the red LED chip presents a prominent emission band centered around 660 nm, closely matching the EL response, indicating efficient radiative recombination within the active region, consistent with band-edge emission of the AlInGaP-based MQW structure. Overall, the PL spectra capture the intrinsic luminescence characteristics of the LED chips and reveal their correlation with EL measurements, underscoring the potential of PL as a noninvasive means to evaluate the emission properties of LED devices.

**Figure 5:**
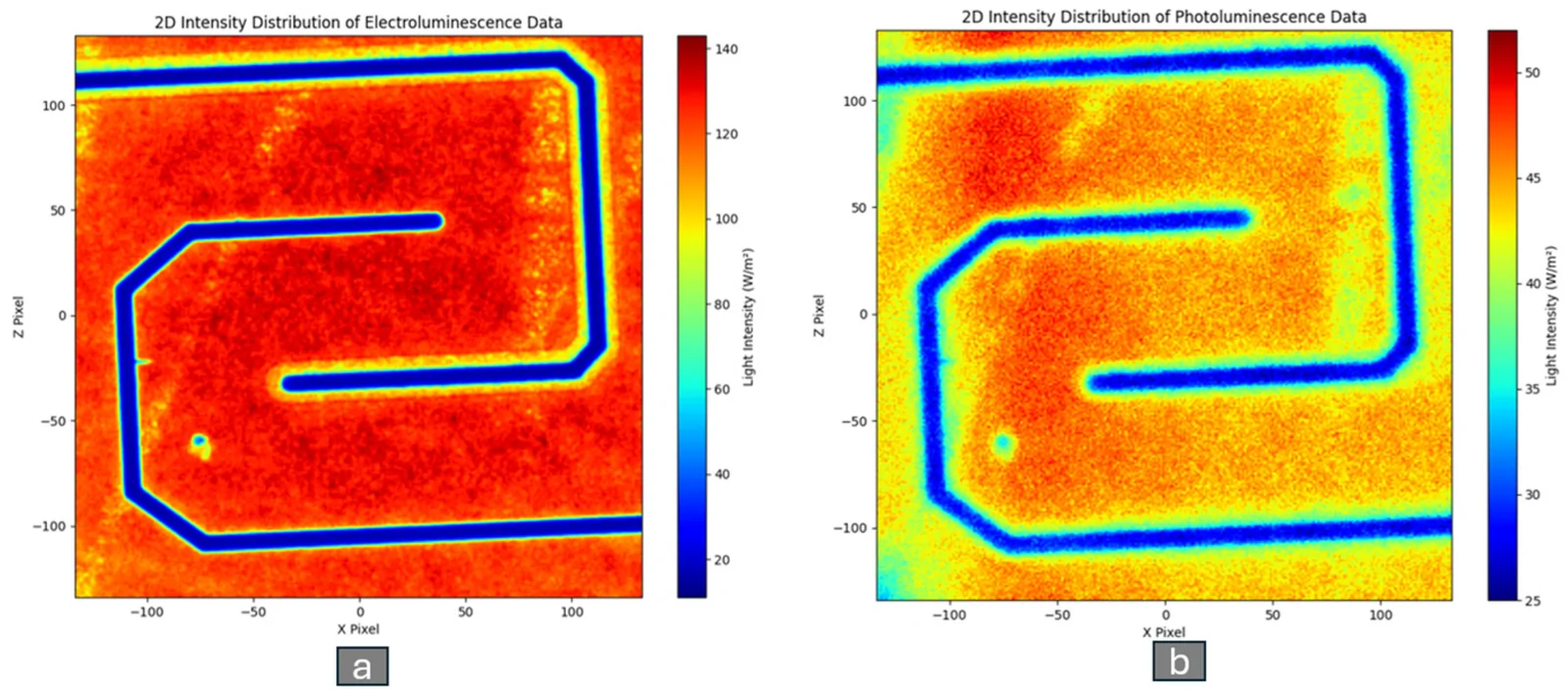
Spatially resolved (**a**) EL and (**b**) PL mapping for the blue LED chip under the normal-collection geometry.

**Figure 6:**
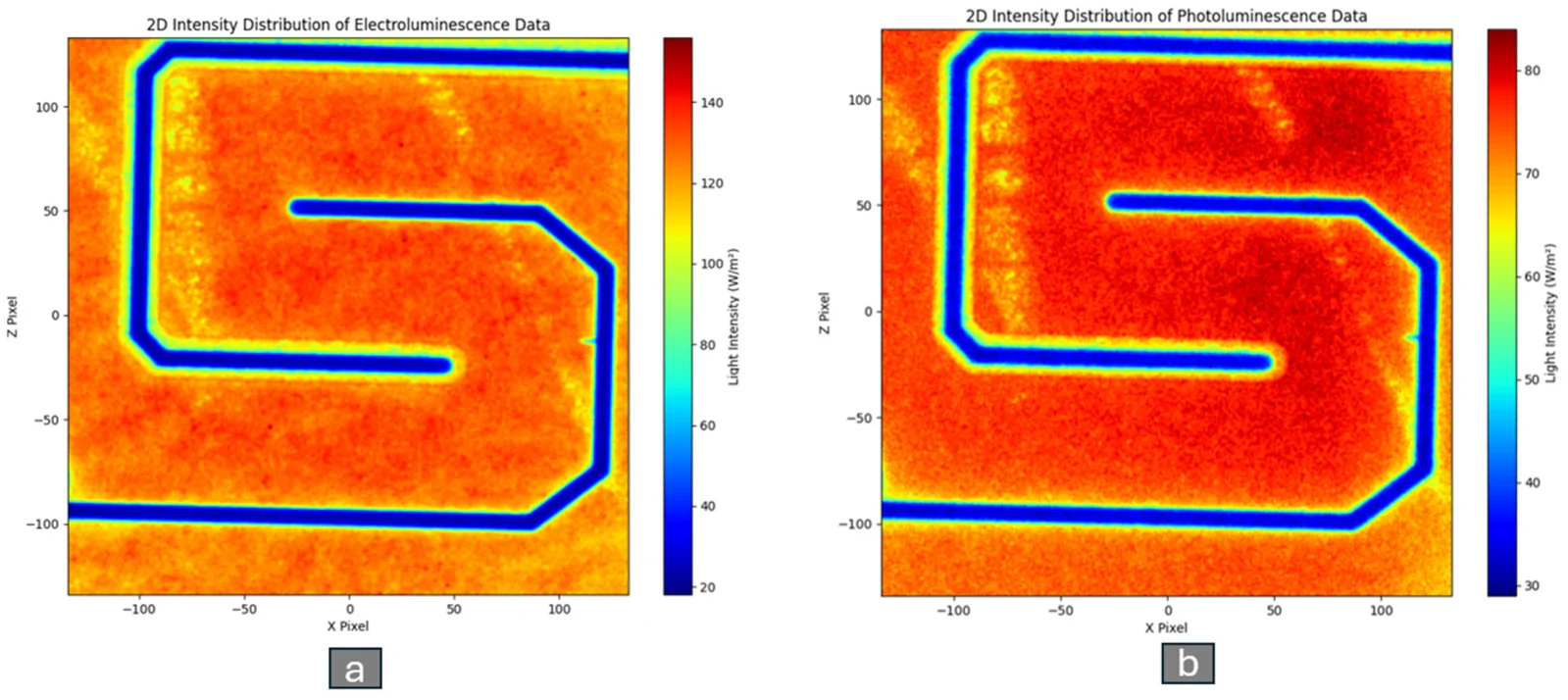
Spatially resolved (**a**) EL and (**b**) PL mapping for the blue LED chip under the oblique-collection geometry.

In this work, we established two dedicated optical setups to enable spatially resolved PL and EL mapping of LED chip samples. These configurations employed high-power LEDs as excitation sources in combination with CCD-based detection, ensuring direct comparability between PL and EL measurements. This approach provided a reliable framework for our evaluation of device emission behavior under both optical excitation and electrical injection. Figure 5 and Figure 6 present the spatially resolved EL and PL mapping results for the blue LED chip under the normal- and oblique-collection geometries, respectively. The EL distribution under normal-incidence conditions in Figure 5a exhibits relatively uniform emission with localized variations, with nonemitting metallic contact regions clearly visible as dark blue areas and a distinct darker spot likely caused by dust contamination. The corresponding PL distribution in Figure 5b reproduces these features with high fidelity, confirming that PL measurements under high-power LED excitation reliably reflected the device’s intrinsic emission characteristics. The EL distribution under oblique-incidence conditions in Figure 6a likewise indicates an overall uniform emission with minor spatial variations. The PL map in Figure 6b captures the same features but with noticeably higher intensity than that observed in the normal-incidence case due to reduced optical loss. Collectively, these results demonstrate that both configurations captured the intrinsic luminescence properties of the blue LED chip and highlight the effects of excitation geometry on the measured PL response.

**Figure 7:**
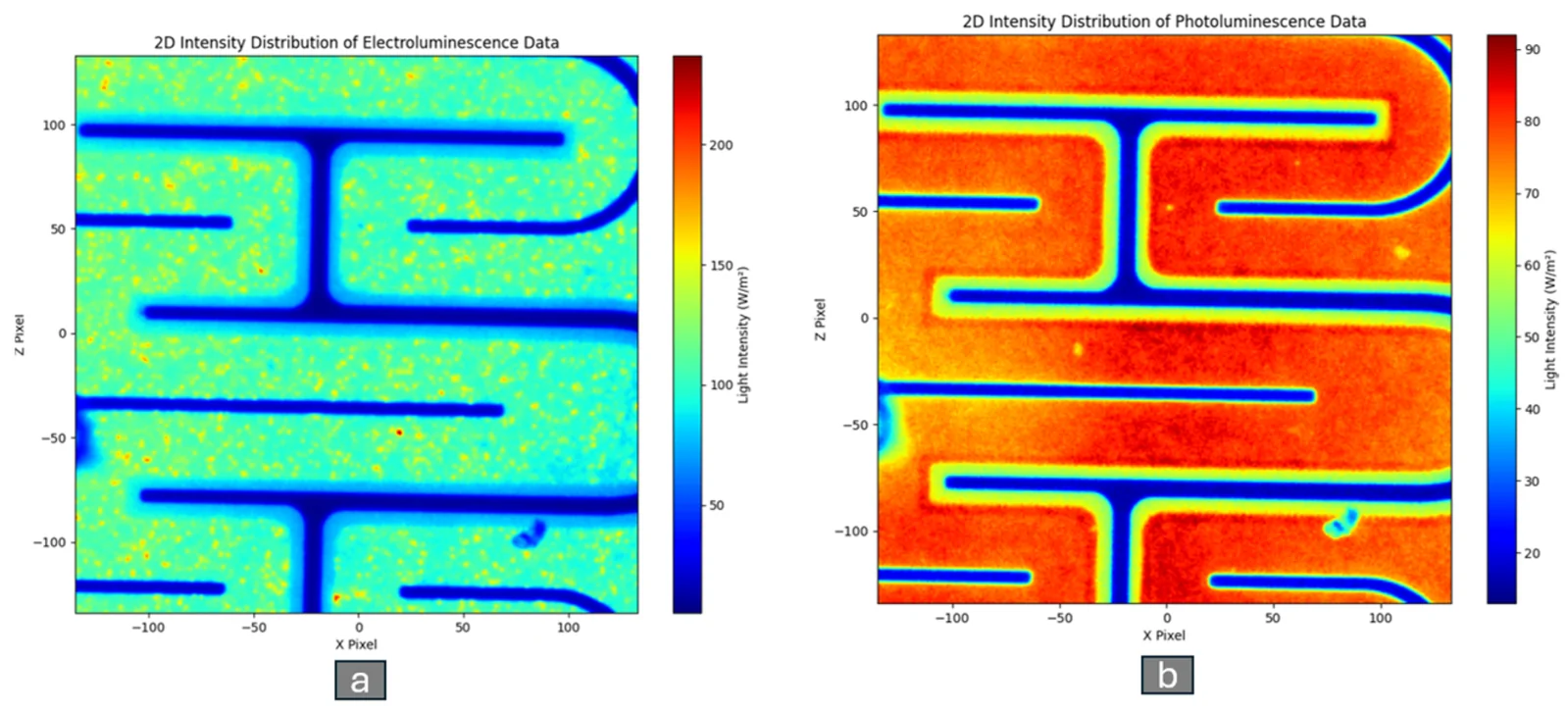
Spatially resolved (**a**) EL and (**b**) PL mapping for the green LED chip under the normal-collection geometry.

**Figure 8:**
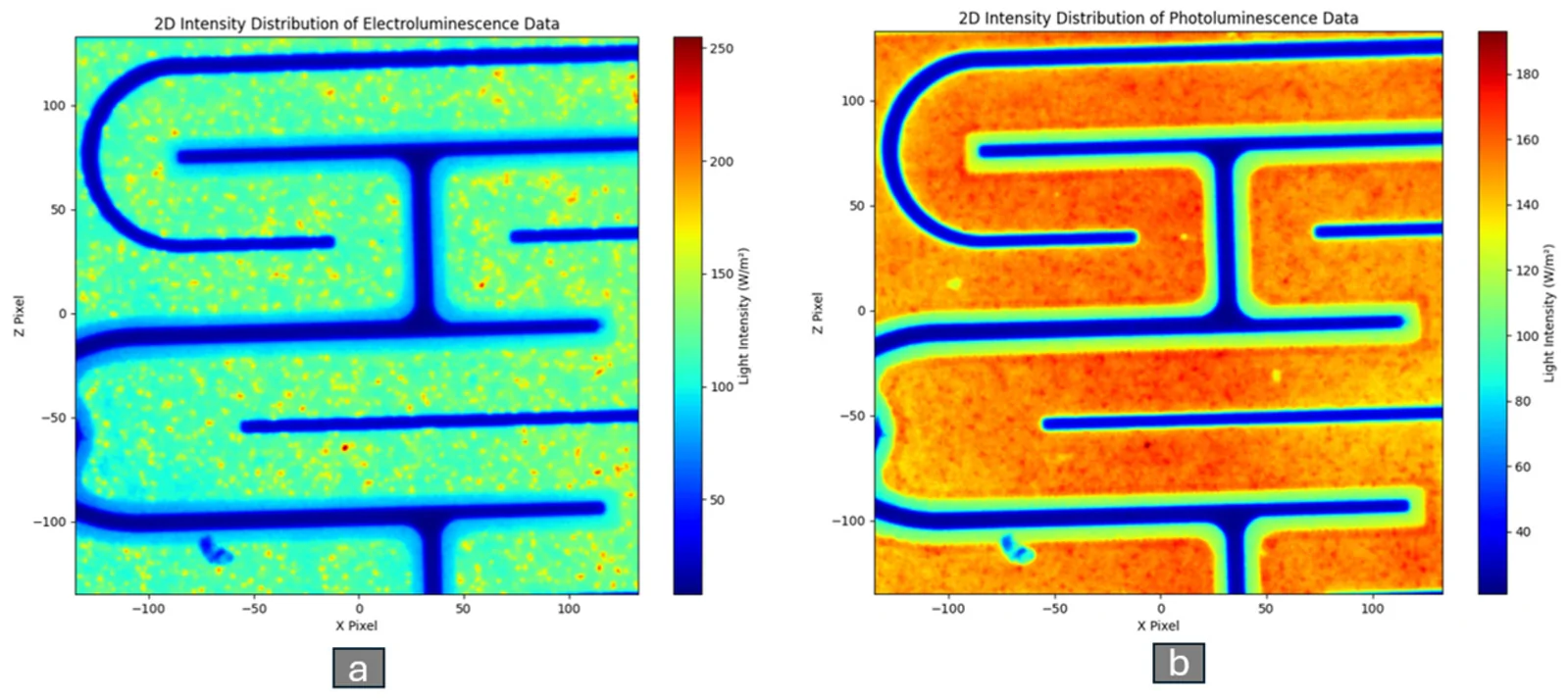
Spatially resolved (**a**) EL and (**b**) PL mapping for the green LED chip under the oblique-collection geometry.

The spatial emission properties of the green LED chip were evaluated under the same measurement conditions. Figure 7 and Figure 8 display the spatially resolved EL and PL mapping results obtained under normal- and oblique-collection geometries. The EL distribution under normal-incidence conditions in Figure 7a exhibits a generally uniform emission profile with localized fluctuations and faint defect-related spots, with the metallic contacts clearly observable as nonemitting regions. The PL map in Figure 7b reproduces these emission features with good fidelity, although attenuation in the normal-incidence optical path reduced map intensity. The EL distribution under oblique-incidence conditions in Figure 8a remains largely uniform with minor spatial variations, whereas the PL map in Figure 8b displays stronger intensity and clearer reproduction of the localized features. These results highlight the role of oblique excitation in strengthening the PL signal and improving the visibility of subtle emission variations in the green LED chip.

**Figure 9:**
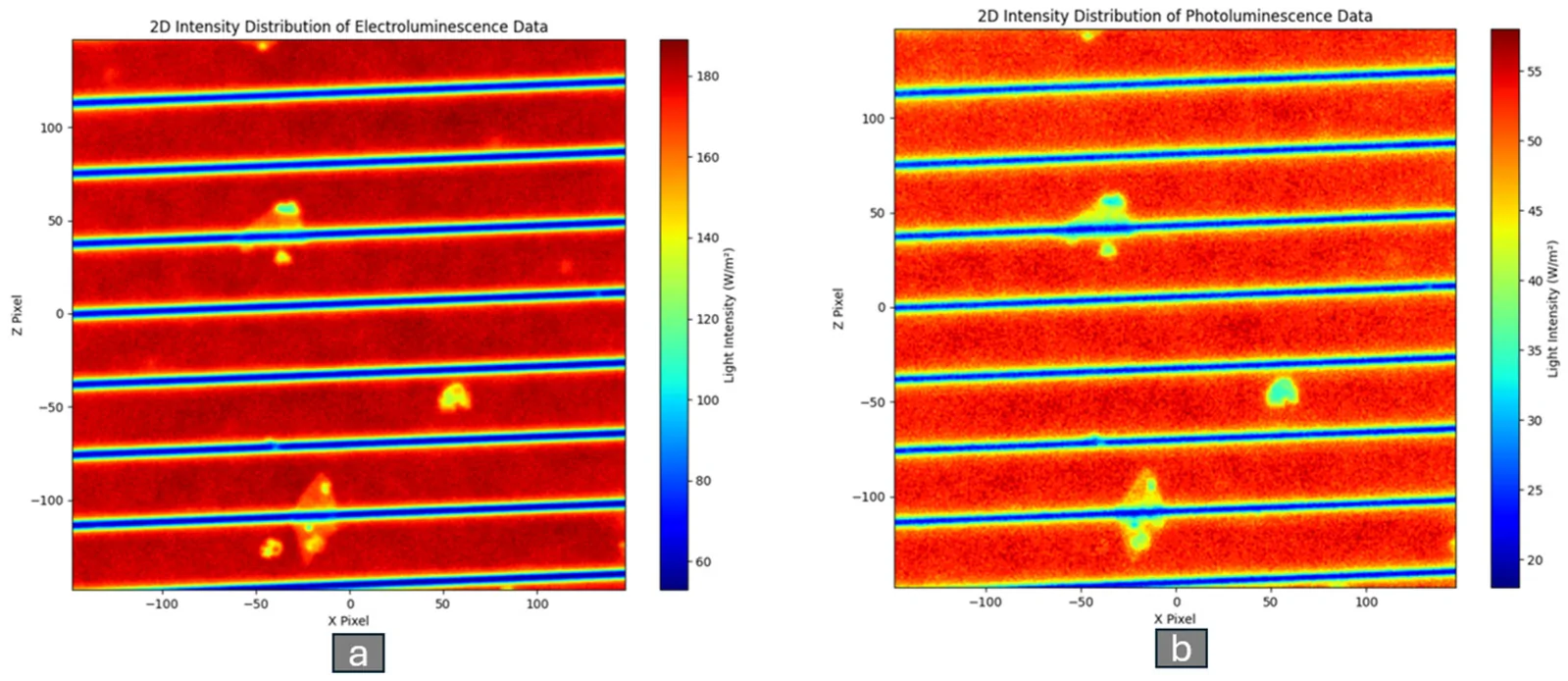
Spatially resolved (**a**) EL and (**b**) PL mapping for the red LED chip under the normal-collection geometry.

**Figure 10:**
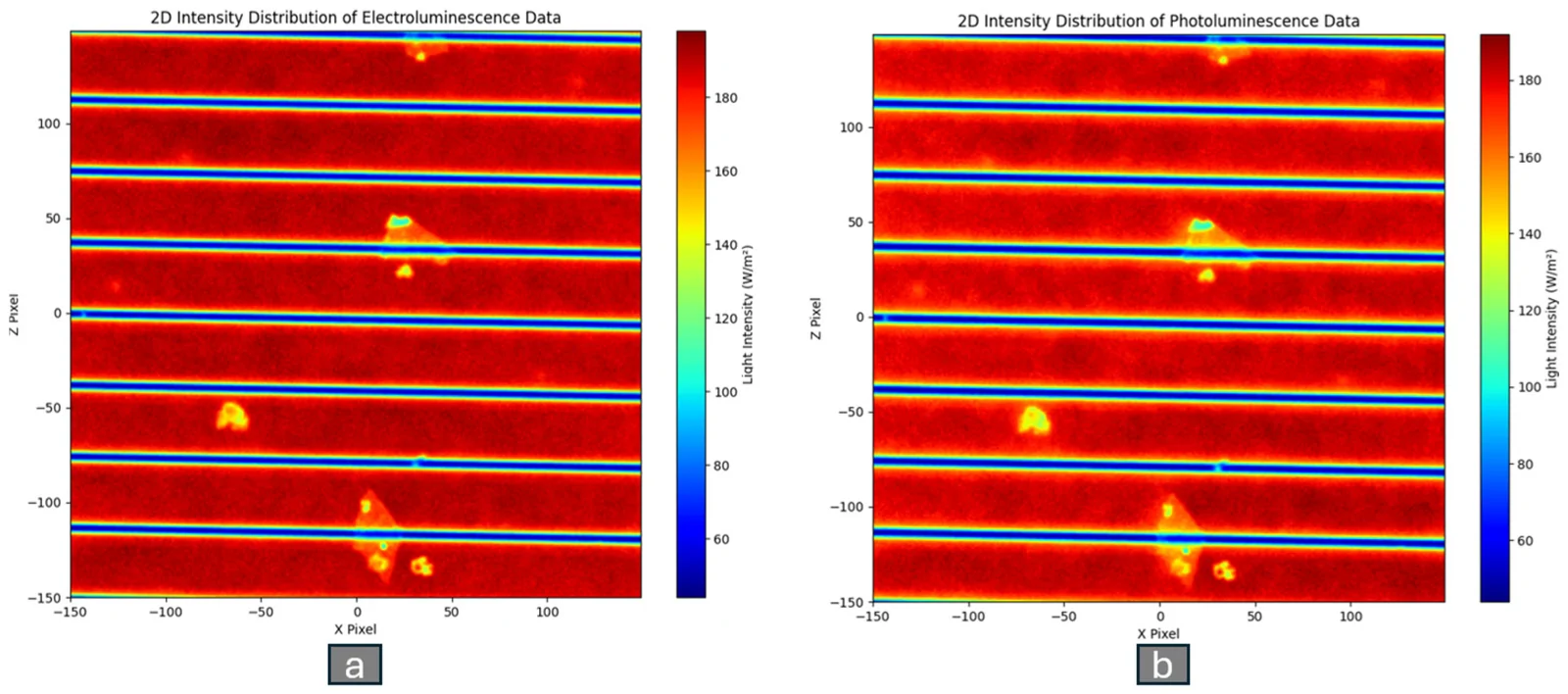
Spatially resolved (**a**) EL and (**b**) PL mapping for the red LED chip under the oblique-collection geometry.

Finally, we examined the emission characteristics of the red LED chip under both configurations. Figure 9 and Figure 10 present the spatially resolved EL and PL mapping results under normal- and oblique-incidence excitation. The EL map under normal-incidence conditions in Figure 9a reveals strong emission across the chip surface with distinct bright and dark regions, reflecting localized variation in radiative efficiency. The corresponding PL distribution in Figure 9b closely follows these patterns, although the overall intensity is weakened by optical loss in the setup. The EL distribution under oblique-incidence conditions in Figure 10a exhibits similar emission features. The PL distribution in Figure 10b not only faithfully reproduces these features but also displays considerably higher intensity than does the normal-incidence distribution. The PL signal enhancement improved the clarity of emission nonuniformities, confirming the advantage of angled excitation for the detection of localized anomalies. Overall, we observed the strongest PL–EL correspondence among the three devices for the red chip, with oblique incidence yielding superior measurement fidelity.

To further evaluate the correspondence between EL and PL mapping results, we performed a pixel-by-pixel correlation analysis for each LED chip under both normal- and oblique-collection geometries. For EL spatial characterization, the driving current was set to 0.15 A (150 mA), which corresponds to the standard nominal operating current for the tested 1000 µm × 1000 µm LED chips prior to packaging. The linear correlation coefficient (R^2^) was employed as a quantitative measure of how well the PL intensity distribution reproduced the spatial emission profile observed in the EL mapping. R^2^ is a widely used statistical parameter that represents the fraction of variance in one dataset (PL) that can be explained by another dataset (EL), making it suitable for assessment of spatial similarity between mapping techniques. An R^2^ value close to 1 was considered to indicate the strong correspondence and high reliability of PL as a substitute for EL in spatial emission characterization. Similar correlation-based approaches have been reported in recent studies, in which EL–PL correlations were used to confirm the structural origin of luminescence variations in GaN-based LEDs [1] and regression analysis of PL/EL images was applied for chip-level performance evaluation in microLED displays [7]. It is worth noting that the primary motivation for calculating R^2^ in this study is to quantitatively evaluate whether the spatial emission pattern captured by wide-field optical excitation (PL) can reliably reproduce the spatial non-uniformity and defect distribution observed under electrical injection (EL), thereby supporting PL as a non-destructive spatial screening tool prior to device packaging. The high R^2^ values observed across different chip structures demonstrate that the proposed LED-PL mapping platform captures dominant spatial non-uniformities and localized non-radiative defect zones with high fidelity, confirming its suitability for rapid pre-packaging chip screening without the need for complex laser-scanning setups or destructive electrical probing.

**Figure 11:**
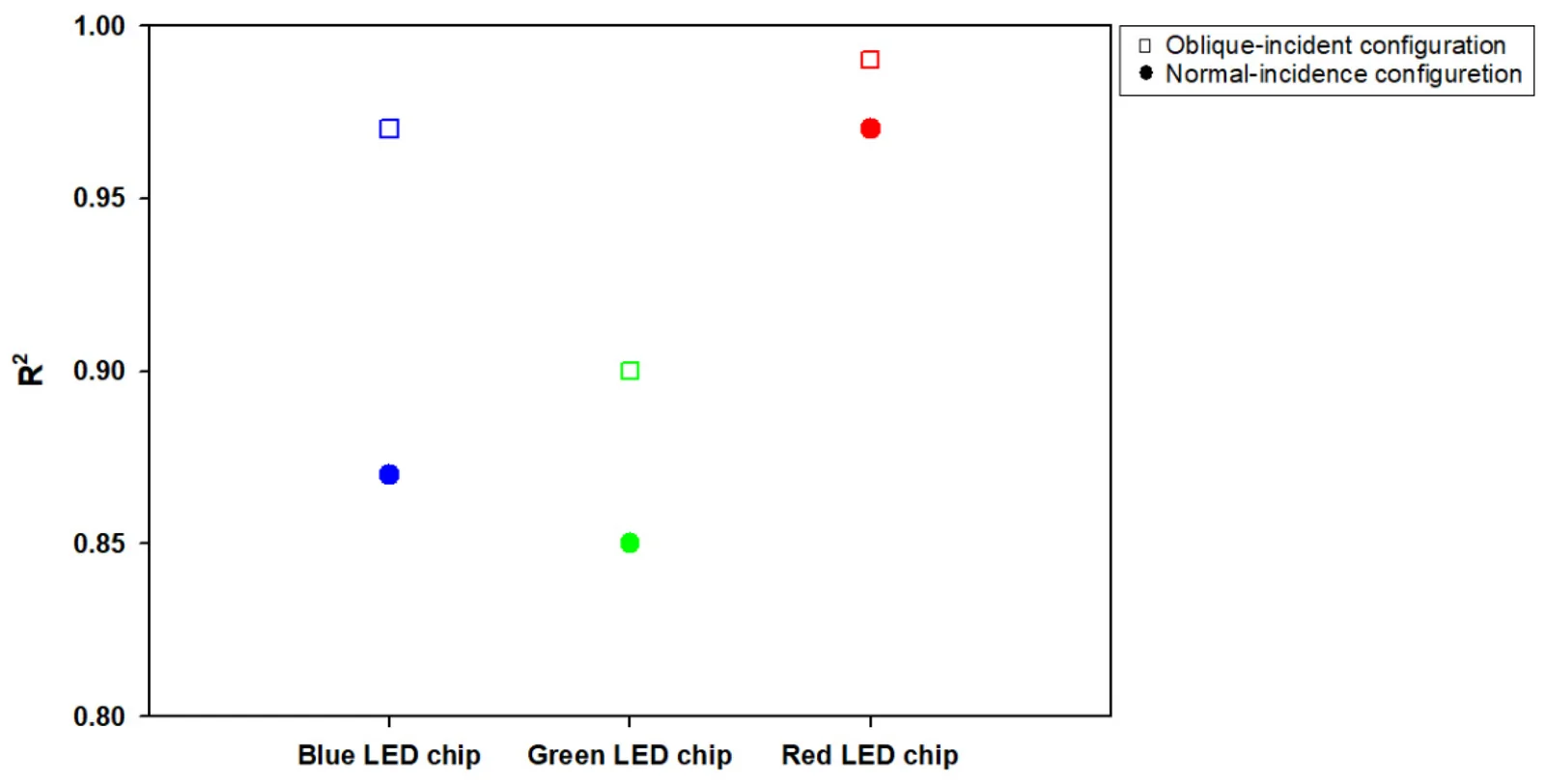
R^2^ values for the blue, green, and red LED chips under normal- and oblique-collection geometries.

Figure 11 summarizes the R^2^ values for the blue, green, and red LED chips under both measurement geometries. For the blue chip, the R^2^ increased from 0.87 under normal incidence to 0.97 under oblique incidence. A similar trend was observed for the green chip, with the R^2^ increasing from 0.85 to 0.90. The red chip had the strongest correlation, with R^2^ values of 0.97 under normal incidence and 0.99 under oblique incidence. The stronger PL–EL spatial correlation observed for the red LED chip (R^2^ = 0.99) compared to the blue and green devices is primarily attributable to quantum-well electro-optic dynamics and optical propagation characteristics. Blue and green InGaN/GaN quantum wells inherently experience a strong Quantum-Confined Stark Effect (QCSE). The external bias applied during EL operation screens the internal piezoelectric field and reshapes the energy band diagram, introducing subtle spatial wave-function shifts compared to the zero-bias PL state and modestly reducing R^2^. Furthermore, the longer emission wavelength of the red chip (~650–670 nm) suffers lower optical self-absorption and reduced interfacial scattering through the transparent contact layer, maintaining higher spatial contrast and gradient fidelity for pixel-level matching.

In all cases, the oblique-collection geometry was associated with higher correlation values than the normal-collection geometry, confirming that angled excitation increases the fidelity of PL relative to EL. The highest R^2^ values under both configurations among all devices were observed for the red chip, indicating the strongest PL–EL correspondence. The superior performance of the oblique-collection geometry is attributable to optical loss reduction and stray-light suppression along the measurement path. In the normal-collection geometry, the beam splitter repeatedly attenuates both the incident excitation light and the emitted PL signal (T = 25%, corresponding to a 75% power loss), which diminishes the PL intensity collected by the CCD and lowers image contrast. Furthermore, the normal-collection geometry shares a coaxial optical path, making it susceptible to specular reflections and scattered excitation light from electrode structures and packaging surfaces. In contrast, the oblique-collection geometry spatially decouples the excitation and collection paths, effectively directing specular reflections away from the collection aperture and reducing stray-light noise. Because the excitation wavelength remains identical, the impact of incident angle on excitation depth is secondary compared to the substantial reduction in optical attenuation and stray light. Consequently, the oblique configuration provides cleaner spatial PL distributions, enabling subtle localized emission anomalies to be resolved more clearly.

Although commercial LED chips were evaluated in this study, high-resolution spatial intensity mapping clearly resolved localized non-uniformities, such as intensity variations near electrode edges and localized defect-induced quenching zones. The strong pixel-level R^2^ correlation between PL and EL maps confirms that the platform possesses high spatial sensitivity (2.4 μm pixel pitch) to detect intrinsic micro-scale non-uniformities. To quantitatively evaluate spatial similarity without intensity scale bias across different excitation modes and geometries, all spatial PL and EL matrices were linearly normalized to 0–1 relative intensity scale. Because Pearson’s correlation coefficient (R^2^) is scale-invariant under linear scaling, this intensity normalization isolates spatial relative contrast and localized defect patterns without altering the calculated R^2^ values. It is important to emphasize that R^2^ serves as a quantitative shape- and contrast-matching metric that mirrors the localized spatial distribution of radiative recombination and pinpoints non-radiative quenching zones under actual electrical operation. Although only representative results are presented in this work, repeated measurements performed on the same chip and on multiple devices of the same type yielded consistent spatial distributions and comparable PL–EL correlation trends, demonstrating the good repeatability and stability of the proposed measurement platform. Combined with the reduced optical attenuation and stray-light suppression discussed above, this repeatability confirms that the oblique-collection geometry provides a particularly reliable configuration for detailed analysis of spatial emission properties in commercial LED chips. This non-destructive, spatially resolved screening capability, together with the platform’s demonstrated repeatability, provides a solid foundation for scaling up to future wafer-level screening applications.

The selection of excitation sources and long-pass filters is also crucial in PL mapping of LED chips with different emission wavelengths. If the excitation wavelength is too close to the cut-off wavelength of the long-pass filter, the excitation light may not be completely blocked, potentially introducing unwanted signal components into the detected PL and thereby affecting the measurement results. Therefore, careful consideration of the wavelength relationship between the excitation source and the optical filter is necessary when high-power LEDs are employed as excitation sources for PL mapping of commercial LED chips. Such consideration can ensure accurate and reliable chip characterization.

## 4. Conclusions

This study proposes a novel and practical research route for spatially resolved photoluminescence (PL) mapping of commercial LED chips by employing high-power LEDs as excitation sources, offering an effective alternative to conventional laser-based PL systems. Compared with traditional approaches that rely on lasers and complex optical instrumentation, the proposed platform provides a cost-effective, compact, and scalable solution with a significantly larger illumination area, making it well suited for inspection for both chip- and wafer-scale applications. By systematically comparing PL and EL mappings, we demonstrate a strong spectral and spatial correlation between optically and electrically driven emission, confirming that steady-state PL excited by high-power LEDs can faithfully reflect intrinsic radiative recombination characteristics and localized nonuniformities in LED chips. A key advantage of the proposed approach is the oblique-collection geometry, which effectively enhances PL signal intensity and spatial fidelity while reducing reflection-related losses, resulting in improved agreement with EL measurements compared with normal-collection geometry. Overall, this LED-based PL mapping platform represents a noninvasive, low-complexity, and industrially relevant characterization methodology that bridges optical and electrical inspection, providing a promising pathway for rapid quality screening, process monitoring, and preliminary spatial performance screening of LED chips and related optoelectronic devices. While the present platform demonstrates strong spatial correspondence between PL and EL distributions for defect and non-uniformity screening, quantitative calibration between spatially integrated PL intensity and absolute EL radiant output or efficiency, which is required to extend this platform toward brightness-based binning or efficiency prediction, remains an important direction for future investigation across a larger and statistically representative chip sample set.

## Figures and Tables

**Table 1:** The specifications of commercial LED chips used in this study.

Color	Peak Wavelength (nm)	Dimension (μm)
Blue	450	968 × 968
Green	535	965 × 965
Red	659	1066 × 1066

## Data Availability

Data are contained within the article.

## References

[1] Yapparov R., Tak T., Ewing J., Nakamura S., DenBaars S.P., Speck J.S., Marcinkevičius S. (2024). Properties of V-defect injectors in long wavelength GaN LEDs studied by near-field electro- and photoluminescence. J. Appl. Phys..

[2] Zimmermann F., Beyer J., Beyer F.C., Gärtner G., Gamov I., Irmscher K., Richter E., Weyers M., Heitmann J. (2021). A Carbon Doping Related Luminescence Band in GaN Revealed by Below Bandgap Excitation. J. Appl. Phys..

[3] Geng X., Liu Y., Zou X., Johansson E., Sá J. (2023). Transient Energy Resolved Photoluminescence Study of Excitons and Free Carriers on FAPbBr_3_ and FAPbBr_3_/SnO_2_ Interfaces. J. Phys. Chem. C.

[4] Levine I., Menzel D., Musiienko A., MacQueen R., Romano N., Vasquez-Montoya M., Unger E., Mora Perez C., Forde A., Neukirch A.J. (2024). Revisiting Sub Band Gap Emission Mechanism in 2D Halide Perovskites: The Role of Defect States. J. Am. Chem. Soc..

[5] Novikov S.A., Valueva A.D., Klepov V.V. (2024). Band Gap Engineering and Photoluminescence Tuning in Halide Double Perovskites. Dalton Trans..

[6] Otto I., Mounir C., Nirschl A., Pfeuffer A., Schäpers T., Schwarz U.T., von Malm N. (2015). Micro pixel light emitting diodes: Impact of the chip process on microscopic electro and photoluminescence. Appl. Phys. Lett..

[7] Chiang H.-Y., Chen S.-A., Chou J.-J., Lin K.-H., Chen Y.-H., Shih C.-S., Huang J.-J. (2024). Chip-level mass detection for micro-LED displays based on regression analysis and deep learning. Opt. Express.

[8] Hong Z., Sun J., Bao T., Zeng Y. (2024). Study of Predicting the Performance of I–V Curves Through Photoluminescence Spectral Characteristics. IEEE Photonics J..

[9] Kim J., Jeong H., Choi W.-J., Jung H. (2021). Probeless Estimation of Electroluminescence Intensities Based on Photoluminescence Measurements of GaN Based Light Emitting Diodes. Curr. Opt. Photon..

[10] Zhang Z., Ishii R., Shojiki K., Funato M., Iida D., Ohkawa K., Kawakami Y. (2022). Correlative Micro-Photoluminescence Study on Hybrid Quantum-Well InGaN Red Light-Emitting Diodes. Phys. Status Solidi B.

[11] Yapparov R., Wong M.S., Tak T., DenBaars S.P., Speck J.S., Marcinkevičius S. (2025). Origin of reduced efficiency in GaN-based micro-LED studied by scanning near-field optical microscopy. Appl. Phys. Lett..

[12] Kim M.K., Choi S., Lee J.-H., Park C.-H., Chung T.-H., Baek J.H., Cho Y.-H. (2017). Investigating carrier localization and transfer in InGaN/GaN quantum wells with V-pits using near-field scanning optical microscopy and correlation analysis. Sci. Rep..

[13] Kim J., Kim H.T., Kim H., Kang S.B., Jeong H., Jung H. (2015). Properties of Defective Regions Observed by Photoluminescence Imaging for GaN Based Light Emitting Diode Epi Wafers. J. Opt. Soc. Korea.

[14] Pantova D.H. (2019). UV LED System for PL Measurements on GaN Samples. Macalester J. Phys. Astron..

[15] Li J., Chen D., Li K., Wang Q., Shi M., Cheng C., Leng J. (2021). Carrier dynamics in InGaN/GaN-based green LED under different excitation sources. Crystals.

